# A Natural Depsipeptide Antibiotic that Targets the E site of the Bacterial Ribosome

**DOI:** 10.21203/rs.3.rs-6925047/v1

**Published:** 2025-07-03

**Authors:** Gerard Wright, Manpreet Kaur, Dmitrii Travin, Max Berger, Manoj Jangra, Martino Morici, Haaris Ahsan Safdari, Dorota Klepacki, Wenliang Wang, Michael Cook, Sommer Chou, Allison Guitor, Kalinka Koteva, Min Xu, Linda Ejim, Lesley Macneil, Nora Vázquez-Laslop, Alexander Mankin, Daniel Wilson

**Affiliations:** McMaster University; McMaster University; University of Illinois at Chicago; University of Hamburg; McMaster University; University of Hamburg; University of Hamburg; University of Illinois at Chicago; McMaster University; M.G. DeGroote Institute for Infectious Disease Research; McMaster University; McMaster University; McMaster University; McMaster University; McMaster University; University of Illinois at Chicago; University of Illinois; University of Hamburg

**Keywords:** Depsipeptide, NRPS, ribosome, antibiotic, E site, translocation, gram-negative bacteria, antimicrobial resistance

## Abstract

A significant challenge in addressing the antibiotic resistance crisis is identifying new antimicrobial compounds. Although natural products produced by fungi and bacteria, particularly actinomycetes, have been the source of most antibiotics discovered over the past 80 years, they have fallen out of favor due to the frequent rediscovery of known drug scaffolds. The current perception is that antibiotic-producing actinomycetes have been over-mined and possess little novelty left to yield. Here, we demonstrate that by using improved fractionation approaches that enrich previously overlooked minor products, even well-studied strains of antibiotic-producing actinomycetes can provide new chemical scaffolds with unique modes of action. By fractionating a library of natural product extracts from soil bacteria, we show that Streptomyces rimosus, the source of the well-known antibiotic oxytetracycline, produces a previously overlooked cyclic depsipeptide antibiotic that we called manikomycin. Manikomycin can kill multi-drug-resistant Enterobacteriaceae and is not susceptible to resistance associated with clinically used antibiotics. Biochemical, genetic, and structural analyses reveal that manikomycin binds in the ‘exit’ or E site of the large subunit of the bacterial ribosome preventing the entry of the 3’ end of the tRNA into the E site and effectively hindering the translocation step of protein synthesis in a sequence context-specific manner. Manikomycin is the first antibacterial agent to target the critical but underexplored E site in the large ribosomal subunit, highlighting its value as a lead for developing new antibiotics.

## Introduction

Microbial natural products, particularly those derived from actinomycetes, have been a dominant source of antibacterial agents over the past eighty years. These antibiotics were identified by testing the ability of crude extracts of their producers to inhibit bacterial growth, a strategy termed the Waksman platform, after microbiologist Selman Waksman, who pioneered this approach^1^. Despite the initial spectacular success of that approach, its utility has decreased over the years due to the frequent re-isolation of common chemical scaffolds and the shift toward target-based high-throughput screens of readily sourced synthetic compounds^2,3^. However, the rise of multidrug-resistant pathogens combined with the lackluster success of target-based screens and synthetic chemical libraries in antibiotic drug discovery has reignited interest in microbial natural products as sources of new antimicrobial agents. The growing realization of the untapped genomic potential in actinomycetes has fueled a renewed interest in these microbes as sources of new drug leads^4^.

Streptomyces genomes show an abundance of biosynthetic gene clusters (BGCs) that potentially encode antibacterial compounds. However, only a small fraction of such compounds have been isolated due to the often low levels of BGC expression, dominance of one of the several produced antibacterials, and insufficiently discriminating analytical approaches. One strategy to tap into the cryptic antibiotic pool is the improved fractionation of natural product extracts^5^, allowing the separation of components with overlapping activities, such as two unrelated antibiotics^6^. We report here the application of this strategy to discover a new antibiotic with a previously unknown mode of action. We identified a novel cyclic depsipeptide antibiotic, manikomycin (MKM), derived from *Streptomyces rimosus*, known since 1950 as a producer of the well-known antibiotic oxytetracycline^7^. We show that MKM exhibits a unique mechanism of action: it binds to the E site of the large subunit of the bacterial ribosome and interferes with translocation in a context-specific manner. Additionally, we found that MKM is effective against drug-resistant Gram-negative pathogens and is unaffected by resistance mechanisms found in bacterial clinical strains, offering a new chemical scaffold for antibiotic development.

## Results

### Identification of MKM

**Figure 1:**
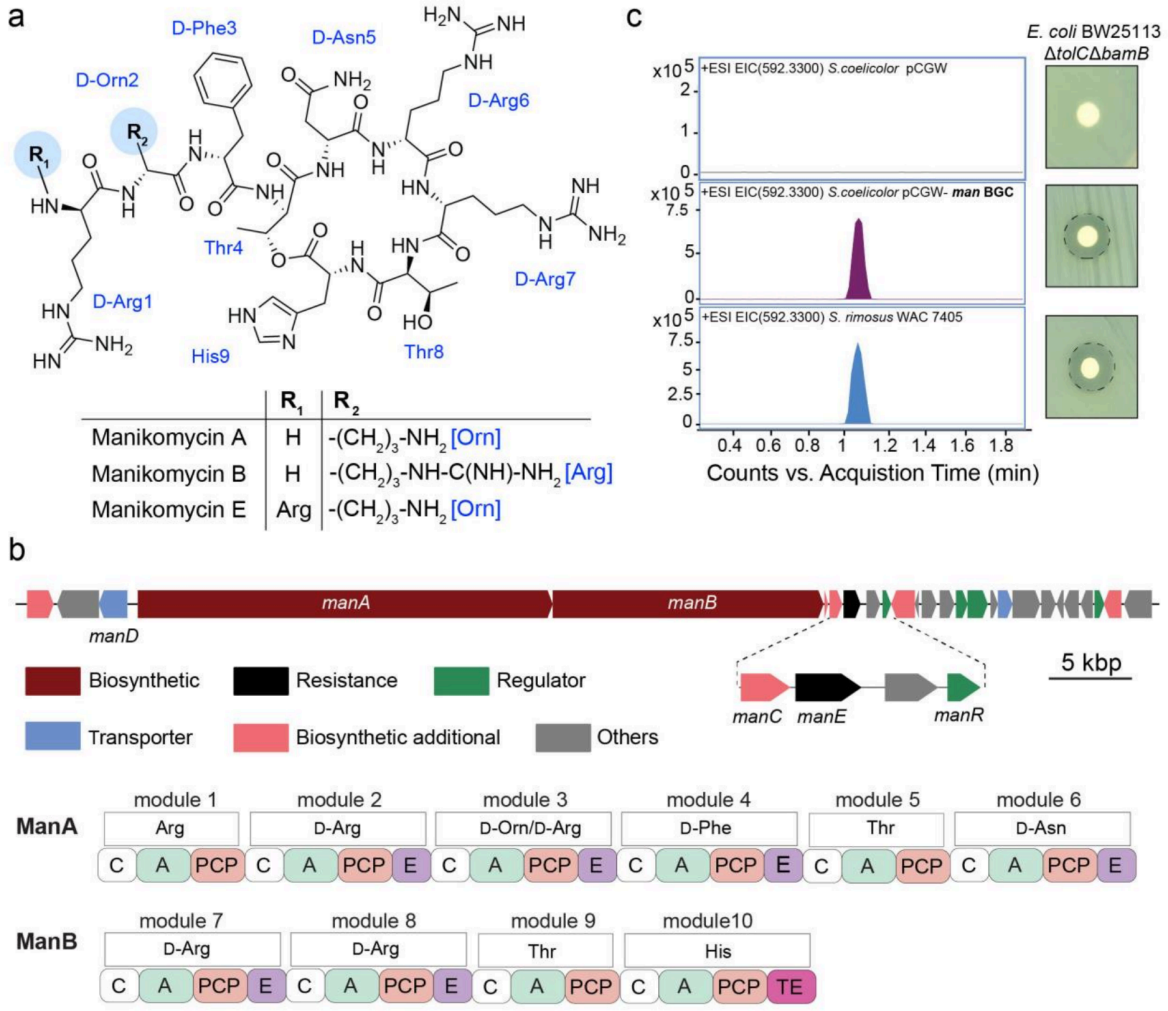
Identification of the cyclic depsipeptide manikomycin (MKM) produced by *Streptomyces rimosus*. **a,** Chemical structure of MKMs. The table represents the substitutions in the various isoforms. Numbering of residues reflects MKM-A structure. **b,**Biosynthetic gene cluster responsible for MKM production (*man* BGC). The predicted functions of select encoded proteins are listed. The modular structure of NRPSs ManA and ManB is depicted below. Domain abbreviations: **A,** adenylation domain; **C,** condensation domain; **PCP,** peptidyl carrier protein; **E,** epimerization domain; **TE,** thioesterase domain **c,** LC-MS analysis and bioactivity of partially purified extracts of MKM conjugants obtained from the heterologous expression of the MKM BGC in *Streptomyces coelicolor*. From top to bottom: (i) Control strain carrying the empty plasmid pCGW. (ii) man BGC expressed from the pCGW plasmid. (iii) Wild-type strain *Streptomyces rimosus* WAC 7405. The doubly charged species of MKM-A observed at 592.83 [M+2H]^2+^, is highlighted in color. The panels on the right display bioactivity assays, where extracts from exconjugants were spotted on the lawn of the indicator strain *E. coli* BW25113 Δ*tolC*Δ*bamB*. The growth inhibition zones are outlined with black dashed circles.

In search of new antibiotics, we screened a collection of methanolic extracts from 255 bacterial strains from our in-house Wright Actinomycetes Collection (WAC) library^6^, primarily consisting of actinomycetes from various soil samples, for growth inhibition of Gram-negative antibiotic-hypersensitive *Escherichia coli* BW25113 Δ*tolC*Δ*bamB*. Fractionation by size exclusion chromatography of the extract from *Streptomyces rimosus*, strain WAC 7405, yielded differentially eluting fractions with antibiotic activity (**Extended Data Fig. 1a**). Global Natural Products Social (GNPS)^8^ molecular networking analysis of the mass spectral signatures of the active fractions identified oxytetracycline and metacycline in fractions 3, 4, and 5 (**Extended Data Fig. 1b**). In contrast, no matches to known compounds were found in active fractions 1 and 2 (**Extended Data Fig. 1c**). Subsequent bioactivity-guided purification of the active compounds from fractions 1 and 2 identified a family of previously unknown cationic cyclic depsipeptides, which we named manikomycins (MKMs). The compound was named from the Hindi and Punjabi word *manik* (“precious gem”), reflecting its rarity and unique mode of action. The most abundant was the nonapeptide manikomycin A (MKM-A, hereafter referred to simply as MKM). The general structure of MKM was determined using a combination of mass spectrometry (MS) and 1D/2D NMR spectroscopy (**Supplementary Figs. 1–8, Supplementary Table 1**) and the stereochemistry of the amino acids was verified by Marfey’s analysis (**Supplementary Fig. 9**). Less abundant MKM variants included the nonapeptide MKM-B, the octapeptides MKM-C and MKM-D, and the decapeptide MKM-E (Fig. 1a, **Supplementary Fig. 10**). MKMs are depsipeptides cyclized through an ester linkage between carboxyl of the C-terminal His and the side chain hydroxyl group of Thr4 (MKM-A numbering) (Fig. 1a). MKM-A, -D, and -E incorporate a D-Orn residue at position 3, while MKM-B and MKM-C have D-Arg in this position (Fig. 1a).

Sequencing of the *Streptomyces* sp. WAC 7405 genome, followed by antiSMASH analysis^9^, identified the putative BGC responsible for the production of MKMs (Fig. 1b, **Supplementary Table 2**). The BGC encompasses the genes encoding the non-ribosomal peptide synthetases (NRPSs) ManA and ManB, consisting of six and four modules, respectively, collectively capable of incorporating ten amino acids into the MKM scaffold. The organization of ManA and ManB modules corresponds to the amino acid composition observed in the MKM-E variant. The final step in MKM biosynthesis involves the macrocyclization between the C-terminal His9 and Thr4 and release from the NRPS complex, which is likely catalyzed by thioesterase (TE) domain encoded by the C-terminal final module^10^ (**Extended Data Fig. 2**). The stereochemistry predicted based on the presence or absence of epimerase domains, accurately aligned with experimental data for all ten amino acids (**Supplementary Fig. 10**).

We transferred the entire 6.7 kb-long MKM BGC into the heterologous host *Streptomyces coelicolor* M1154. The resulting strain successfully secreted MKMs, as confirmed by MS and antibacterial activity assays (Fig. 1c), validating the assignment of the MKM BGC in *Streptomyces* sp. WAC 7405.

### MKM inhibits bacterial translation by targeting the ribosome

**Figure 2:**
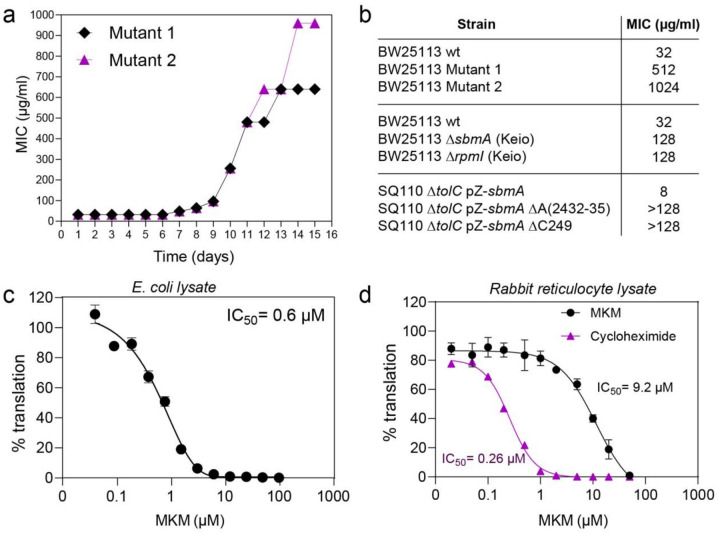
MKM targets bacterial protein synthesis. **a,** Acquisition of resistance during serial passaging in the presence of sub-MIC levels of MKM. The y-axis represents the highest concentration at which cells continued to grow during passaging. **b,** MIC values for the MKM-resistant mutants and corresponding wt strains (*E. coli* BW25113, *E. coli* Δ*sbmA*, Δ*rpmI* and *E. coli*strain SQ110Δ*tolC*::pZ*sbmA*). **c,** MKM inhibits protein synthesis in *E. coli* cell-free transcription-translation system programmed with firefly luciferase-encoding plasmid. % Translation represents residual luminescence compared to the untreated control. Data are presented as mean ±SD (standard deviation) of three technical replicates and are representatives of two biological replicates with similar results. **d,** Inhibition of protein synthesis in rabbit reticulocyte lysate programmed with luciferase mRNA by MKM at various concentrations. Cycloheximide was used as a positive control.

The abundance of positively charged residues in MKM could indicate membranolytic activity, similar to other cationic antimicrobial peptides. However, MKM failed to permeabilize or distrupt the bacterial membrane (**Extended Data Fig. 3a,b**), and no detectable changes in bacterial cell morphology were observed that would indicate membrane restructuring (**Extended Data Fig. 3c**). Therefore, we shifted our focus to the possibility that MKM acts upon an intracellular target. Passaging of antibiotic *E. coli* strain BW25113^11^ in the presence of increasing concentrations of MKM led to the selection of highly resistant MKM mutants with the minimum inhibitory concentration (MIC) of 512–1024 μg/ml, exceeding the MIC of the parental strain by 16- to 32-fold (Fig. 2a,b). Whole genome sequencing of one of the resistant mutants identified, among other changes, loss-of-function mutations in the gene *sbmA* encoding a peptide transporter of the inner membrane involved in the uptake of other peptide antibiotics^12–14^ (**Supplementary Table 3**). In general agreement with this finding, the *ΔsbmA* strain from the *E. coli* single-gene knockout Keio collection^15^ exhibited a 4-fold increase in MIC (Fig. 2b). Deletion of the genes encoding the components of another transporter, YejABEF, that also internalizes peptide antibiotics^16,17^, similarly resulted in a 2–8-fold increase in MIC (**Extended Data Fig. 4a-b**), indicating that MKM, unlike some other antibiotics whose uptake depends on a single transporter^18^, exploits several pathways to enter the cell and likely acts upon an intracellular target.

Sequencing of the genome of another selected MKM-resistant mutant showed the presence of a nonsense mutation in the *rpmI* gene encoding the large ribosomal subunit protein bL35 (**Supplementary Table 3**). This finding pointed to the ribosome as a possible cellular target of MKM. Most ribosome-targeting antibiotics interact with rRNA^19^, however, because of the redundancy of rRNA genes in bacteria, such as *E. coli*, rRNA resistance mutations are extremely rare. Therefore, to verify that MKM interferes with bacterial growth by inhibiting the ribosome, we selected MKM resistance mutants in the antibiotic-hypersusceptible *E. coli* strain SQ110Δ*tolC*::pZ*sbmA* that carries a single copy of the rRNA operon on its chromosome and overexpresses the MKM-internalizing transporter^20,21^. Several hundred resistant colonies appeared with a frequency of ~10^−7^ when cells were plated on agar supplemented with 4×MIC of MKM. Sequencing the 23S rRNA gene in 11 randomly picked MKM-resistant mutants revealed a deletion of a single adenine in the four-adenine stretch (A2432-A2435) in 10 of them and a deletion of C249 in the remaining mutant. All these mutants showed >16-fold increase in MKM MIC (Fig. 2b).

Strikingly, these mutations, as well as the bL35 protein, are located near the E site of the bacterial large ribosomal subunit, a location that has never been previously identified as a binding site for antibacterial compounds^22^.

Using an *E. coli* cell-free translation system, we confirmed that MKM is a potent inhibitor of bacterial protein synthesis (IC_50_ = 0.6 μM) (Fig. 2c). In contrast, MKM acted as a much weaker inhibitor of protein synthesis in a rabbit reticulocyte lysate translation assay, exhibiting IC_50_ of 9.2 μM, approximately 15-fold higher than that for the bacterial system and 35-fold higher than that of cycloheximide (Fig. 2d), a known E-site inhibitor of eukaryotic translation^23^. These results reveal MKM as a selective antibacterial compound targeting protein synthesis.

### Binding of MKM to the E site of the large ribosomal subunit

**Figure 3:**
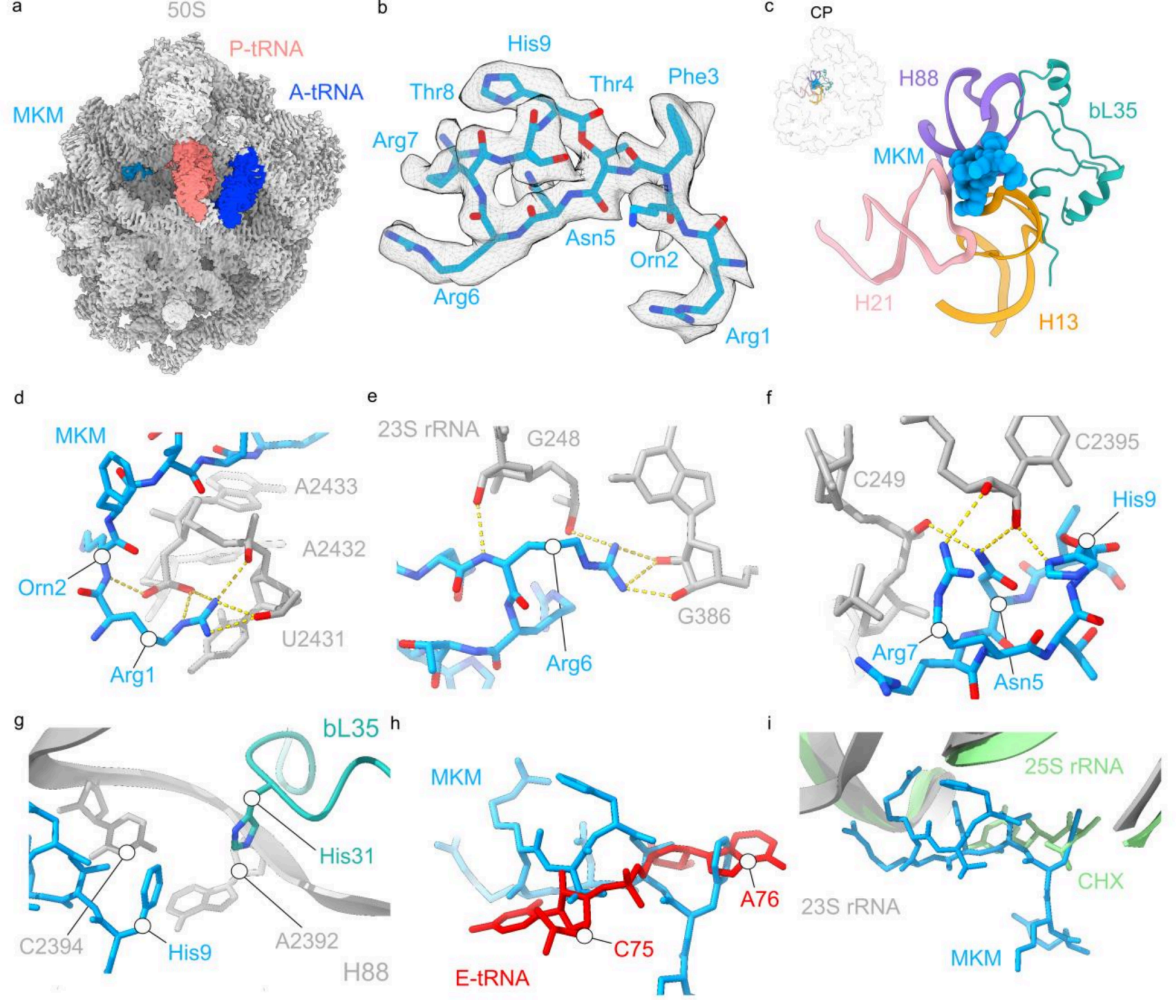
MKM binds within the E-site of the *E. coli* 50S subunit. **a,** Cryo-EM density map of the 50S subunit with A-tRNA (dark blue), P-tRNA (salmon), and MKM (light blue) highlighted. **b,** Cryo-EM density (grey mesh) and molecular model for MKM (blue). **c,** Binding site of MKM on the 50S subunit, encompassing H13 (gold), H21 (pink) and H88 (purple) of the 23S rRNA and ribosomal protein bL35 (green). Inset shows the orientation of MKM binding site on the 50S subunit (transparent grey), with central protuberance (CP) labelled. **d-f,** Potential hydrogen bond interactions (dashed yellow line) between MKM (light blue) and nucleotides of the 23S rRNA (grey). **g,** Ribosomal protein bL35 (green) does not directly interact with MKM (blue), but directly interacts with 23S rRNA nucleotides located within H88 (grey). **h,** Overlay of the binding position of MKM (blue) with the modelled E-tRNA (red) showing clash with A76 and C75 of the CCA-end of the E-tRNA (PDB ID 8AKN)^28^. **i,** Overlay of the binding position of MKM (blue) on the *E. coli* 70S ribosome (23S rRNA, grey) with cycloheximide (green) bound to the eukaryotic yeast 80S ribosome (25S rRNA, lime) (PDB ID 4U3U)^29^.

We used high-resolution structural analysis to determine the binding site of MKM on the translating ribosome. First, by using toeprinting, a technique that allows mapping the position of a drug-arrested ribosome on mRNA^24,25^, we showed that the ribosomes stall primarily on the third codon of the model *ermBL* mRNA translated in an *E. coli in vitro* protein synthesis system in the presence of MKM (**Extended Data Fig. 5a**). We then analyzed the resulting MKM-stalled ribosome complexes (MKM-SRC) using single particle cryo-electron microscopy (cryo-EM), as we have done previously for other antibiotic-SRCs^26,27^. *In silico* sorting of the cryo-EM data revealed one major functional state of the ribosome containing A- and P-tRNAs, but no E-tRNA (**Supplementary Fig. 11**), which could be refined to 2.5 Å resolution (**Supplementary Fig. 12, Supplementary Table 4**), and further improved to 2.4 Å by focused refinement on the 50S subunit (Fig. 3a and **Supplementary Fig. 12, Supplementary Table 4**). After modelling of the 70S ribosome as well as A- and P-site tRNAs, two additional cryo-EM densities remained that could be unambiguously assigned to MKM. One MKM binding site (which we refer to as primary site) is located within the E site of the 50S subunit (Fig. 3a–c). The second binding site is at the back of the 50S subunit (**Supplementary Fig. 13**), far from any known ribosomal functional centers. The *de novo* modelled conformation of MKM confirms that this cyclic peptide is formed by linkage of the sidechain hydroxyl of Thr4 with the backbone carboxyl of His9 and contains a linear N-terminal tail consisting of Arg1, Orn2 and Phe3 (Fig. 3b and **Supplementary Fig. 13**). In the primary binding site, the core ring of MKM inserts into a pocket formed by the tips of the 23S rRNA helices H13 and H21 in domain I and nucleotides at the base of H88 in domain V (Fig. 3c). Many direct hydrogen bonding interactions can be formed between the charged residues of MKM (Arg1, Orn2 within the N-terminal tail and Asn5, Arg6, Arg7 and His9 in the ring), predominantly with the sugar-phosphate backbone of 23S rRNA nucleotides, including G248-C249 (H13), G386-U387 (H21), C2395 and U2431-A2433 (H88) (Fig. 3d–f **and Video S1**). Additionally, a network of water-mediated interactions is observed between Asn5 and Arg7 of MKM with G248 and C249 within H13 (**Supplementary Fig. 14 and Video S1**). Consistent with this site being the primary point of MKM action, the locations of the rRNA resistance mutations (deletion of C249, or of an adenine within the A2432-A2435 stretch) are in close proximity to the bound antibiotic. These deletion mutations likely confer resistance by inducing local conformational changes in the 23S rRNA that disrupt the interactions observed with MKM (Fig. 3d–f and **Supplementary Fig. 14**). Protein bL35, whose truncation by a nonsense mutation confers resistance, is also located close to this primary MKM binding site but does not directly contact the antibiotic. Instead, bL35 establishes multiple interactions with H13 and H88 (Fig. 3c, g) and therefore, MKM resistance resulting from the bL35 gene mutation probably arises due to allosteric perturbations in the MKM binding site via conformational changes in the 23S rRNA (Fig. 3g).

Bound in its primary site, MKM obstructs placement of C75 and A76 of the CCA end of a deacylated tRNA within the E-site of the large ribosomal subunit^28^, with the most significant overlap of Phe3 of MKM with the 3’ terminal adenine of the E-tRNA (Fig. 3h). Noteworthy, the E-site of the large subunit of archaeal and eukaryotic ribosomes is the target of several translation inhibitors, such as cycloheximide (CHX) (Fig. 3i), lactimidomycin (LTM), 13-deoxytedanolide, and phyllanthoside^29–31^, that exhibit overlapping but distinct binding modes compared to MKM (**Extended Data Fig. 6a-h**) but do not inhibit bacterial translation^32,33^. A comparison of the primary MKM binding site with eukaryotic ribosomes^29,30^ shows that the presence of an archaeal/eukaryotic-specific ribosomal protein, eL42, would sterically block binding of MKM to the E-site (**Extended Data Fig. 6i-l**). This explains the low toxicity of MKM against human cell lines (**Extended Data Table 1**) and poor inhibition by MKM of eukaryotic *in vitro* translation (Fig. 2d).

### Mechanism of protein synthesis inhibition by MKM

**Figure 4:**
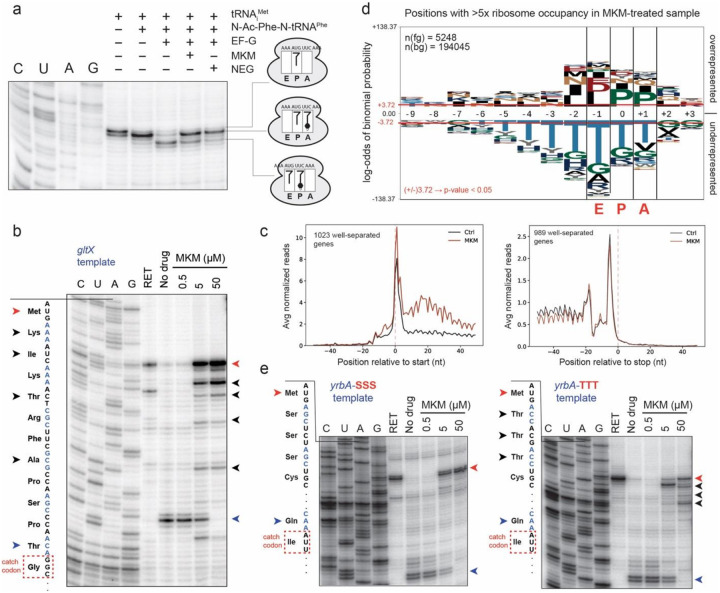
Mechanism of translation inhibition by MKM. **a,** Translocation assay. **b,** Toeprinting analysis of MKM effect on GltX translation. Blue arrowhead indicates ribosome stalling in front of the Gly 13 codon due to the presence of Gly-RS inhibitor (Gly-AMS) in all the reactions. The start codon toeprinting band is indicated by a red arrowhead and the other MKM-induced stalls are marked by black arrowheads. Retapamulin (RET), the known initiation inhibitor^99^, was used as a control. **c,** Metagene analysis of ribosome density around the start and stop codons of the genes in MKM-treated (red line) or untreated (black like) cells deduced from the results of ribosome profiling. **d,** pLogo analysis of codons specifying individual amino acids at the sites of preferential MKM-induced ribosome stalling. **e,** Toeprinting analysis of MKM-induced ribosome stalling during translation of the *yrbA* template carrying the insertion of three Thr codons or three Ser codons after the start codon of the gene. Red and black arrowheads indicate positions of the ribosomes stalled at the start codon or internal codons of the gene, respectively. Blue arrowhead indicates ribosome stalling in front of the Ile 13 codon due to the presence of the Ile-RS inhibitor mupirocin in all the reactions.

The overlap in the binding site of MKM with the CCA end of the E-site tRNA (Fig. 3i), suggests that MKM would impede the translocation of the P-tRNA into the E site (and therefore, of A-tRNA into the P site). Consistently, the majority of MKM-SRCs visualized in the cryo-EM reconstructions were in the pre-translocation state containing A- and P-site tRNAs, whereas no hybrid (A/P- and P/E-tRNAs) or post-translocation (P- and E-tRNAs) states were detected (**Supplementary Fig. 11**). To experimentally verify the mode of MKM action, we directly tested the ability of MKM to interfere with translocation *in vitro* (Fig. 4a). To do this, we generated a pre-translocation complex by binding deacylated tRNA_i_^Met^ in the P site and N-acetyl-Phe-tRNA^Phe^ in the A site of the *E. coli* ribosome. Upon addition of elongation factor G (EF-G), in the absence of antibiotics, the ribosome readily translocated to the next mRNA codon. This reaction, however, was notably inhibited by MKM or the antibiotic negamycin, a known translocation inhibitor^34^.

Our original toeprinting experiments showed that MKM stalled the ribosome on the third codon of the *ermBL* template encoding a protein with the N-terminal sequence MLVFQ (**Extended Data Fig. 5a**). Consistently, careful analysis of the cryo-EM structure of the ribosome stalled by MKM on the *ermBL* mRNA revealed the P- and A-tRNAs as being tRNA^Val^ and tRNA^Phe^, respectively. However, a universal inhibitor of translocation added to an *in vitro* translation reaction prior to (its onset) is expected to arrest the ribosome at the start codon (**Supplementary Fig. 15**). Intrigued by this seeming contradiction, we examined by toeprinting the effect of MKM on translation of a different template, encoding the *E. coli* protein GltX (Fig. 4b). While MKM arrested some ribosomes at the start codon, a notable fraction of them became arrested at several specific sites along the mRNA, suggesting that the antibiotic inhibits translation elongation (likely interfering with translocation, see Fig. 4a), but its action may be affected by the sequence context of the mRNA or the synthesized protein.

To gain further insights into the sequence specificity of MKM action, we analyzed its effect upon translation in *E. coli* cells by ribosome profiling, a technique that reveals the redistribution of ribosomes through the length of mRNAs in response to antibiotic treatment^35,36^. Metagene analysis showed increased ribosome occupancy at the early mRNA codons in the cells exposed to MKM (Fig 4c), an effect commonly observed with elongation-inhibiting antibiotics^37–39^. Analysis of the sites of preferential MKM-induced ribosome stalling showed enrichment of Pro codons in the P and A sites of the drug-arrested ribosome (Fig. 4d). Because incorporation of prolines into the synthesized proteins is generally problematic^40–42^, it is possible that the resulting transient idling of a ribosome translating Pro codons offers more time for the E-site tRNA to dissociate prior to the subsequent translocation, thereby increasing the accessibility of the vacant E site for MKM binding. We also noted increased presence of specific amino acid residues in the three C-terminal positions of the nascent peptide at the preferential sites of MKM action (most notably Ile, Leu and Pro), suggesting nascent polypeptide-dependent ribosome pausing that could also facilitate MKM binding. Interestingly, the strongest effect was exclusion of Thr at the sites of MKM-induced arrest and over several preceding codons (Fig 4d). This result suggests that the ribosome translating through threonine codons is more impervious to antibiotic action than at other codons. To experimentally verify this observation, we modified the *yrbA*-derived template (**Extended Data Fig. 5b**) used in our previous studies^43^ by introducing either three Thr codons (*yrbA*-TTT mRNA) or, as a control, three Ser codons (that did not show enrichment or depletion at the sites of MKM stalling) after the mRNA start codon (*yrbA*-SSS). In agreement with the observations drawn from ribosome profiling, at high concentrations of MKM, no ribosomes pass downstream of the start codon of the *yrbA*-SSS template (Fig. 4e, left gel). In contrast, despite the presence of MKM, a fraction of ribosomes was able to move through the Thr codons of the *yrbA*-TTT mRNA (Fig. 4e, right gel). We envision that either the structural properties of tRNA^Thr^ or peculiarities of its translocation may account for the context specificity of the MKM action.

### Mechanisms of MKM self-resistance in *S. rimosus*

**Figure 5:**
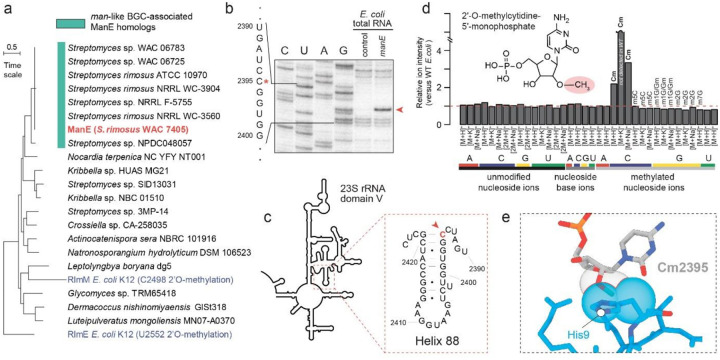
ManE methyltransferase confers resistance to MKM by modification of 2’OH group of C2395 in 23S rRNA. **a,** Phylogenetic tree of ManE homologs. Close homologs found in *man*-like BGCs are highlighted in green. Distant homologs not associated with any BGC are also displayed. *Escherichia coli* RlmE served as the outgroup. **b,** Primer extension analysis of 23S rRNA modification. Note the appearance of a band reflecting the modification of C2395 for an RNA sample from ManE-expressing, but not control *E. coli*. **c,** Location of C2395 in the 2D structure of *E. coli* 23S rRNA. **d,** Bar graphs depicting integrated ion intensities for individual ions, including nucleosides, nucleobases resulting from in-source fragmentation, and methylated nucleosides, expressed as a ratio relative to the corresponding ions in WT strains. Ions are annotated by the molecular position of methylation, determined by the retention time of individual standards. The chemical structure of 2’-O-methylcytidine-5’monophosphate is shown as an inset. **e,** Modelling of the 2’-O-methyl group of Cm2395 shows that it sterically clashes with the side chain of His9 in MKM.

In order to avoid self-toxicity, bacterial antibiotic producers must be immune to the action of the inhibitor they synthesize. Such immunity to ribosome-targeting antibiotics is often conferred by methylation of specific rRNA residues located at the sites of the compounds action^44–46^. Analyzing genes associated with the MKM BGC, we identified a putative rRNA methyltransferase gene, *manE* (Fig. 1b), which we hypothesized might encode a self-resistance-conferring protein. Notably, the *manE* gene is found exclusively in the strains of *Streptomyces rimosus* whose genomes encode MKM BGCs, underscoring its relevance for MKM production and action (Fig. 5a, **Supplementary Fig. 16**). To investigate the putative site of ManE action and its role in MKM resistance, we expressed *manE* gene in *E. coli*. ManE expression conferred resistance to MKM (>32-fold increase in MIC in *E. coli* BW25113 D*tolC*D*bamB*) (**Extended Data Fig. 7a,b**), but not to other translation inhibitors (**Extended Data Fig. 7c-j**), suggesting that ManE modifies rRNA within the site of MKM action. Primer extension on rRNA isolated from WT or ManE-expressing *E. coli* was used to identify the site(s) of ManE-installed rRNA modification. A cDNA band corresponding to the reverse transcriptase pausing in front of C2395 in ManE-modified 23S rRNA suggested the presence of a ManE-catalyzed posttranscriptional modification of this nucleotide (Fig. 5b,c). To verify the nature of the modification, the 23S rRNA from WT and ManE-expressing *E. coli* cells was isolated, digested to nucleosides, and analyzed by Hydrophilic Interaction Liquid Chromatography-Mass Spectrometry (HILIC-MS)^47^. The specific ions present at increased abundance in the ManE-expressing strain were consistent with methylation of the 2’-OH of the cytidine ribose (Cm) (Fig. 5d). Noteworthy, in our structure, the 2’OH of C2395 directly interacts with MKM in its primary binding site, and methylation of the 2’OH of C2395 would preclude the formation of this hydrogen bond with MKM, thus providing a structural explanation as to how methylation confers MKM resistance (Fig. 5e).

### Antimicrobial efficacy of MKM

**Figure 6:**
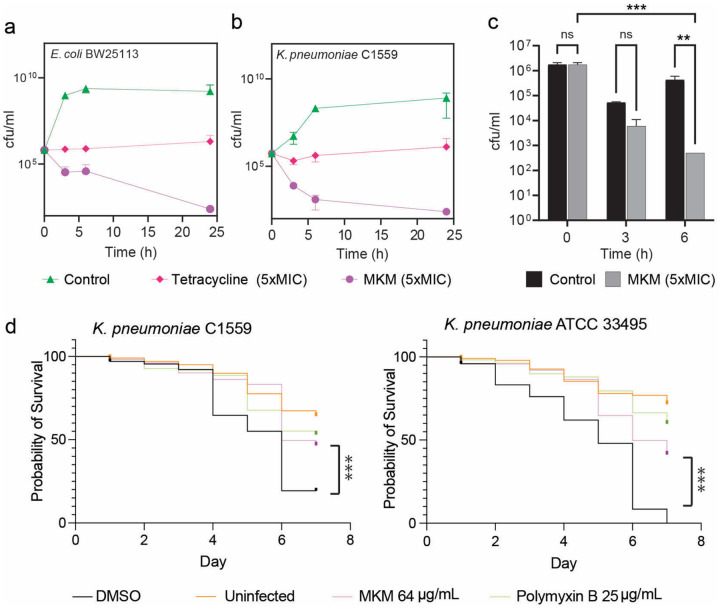
Antimicrobial activity of MKM. **a, b,** *In vitro* time-kill assay in MHB medium showing the bactericidal effect of MKM against *E. coli* BW25113 and *K. pneumoniae* C1559, with tetracycline as a positive control. Data represent the mean of three biological replicates, with error bars indicating standard deviation (SD). **c,** Effect of MKM in an *ex vivo* human blood model. *K. pneumoniae* C1559 was inoculated in human blood, and bacterial CFU were enumerated after 3 and 6 hours of treatment with MKM. Data are plotted as mean ± SD from three biological experiments. Statistical significance was determined using two-way ANOVA (***P ≤ 0.001; **P < 0.01; ns, non-significant, p>0.05). d, Survival curves of *C. elegans* infected with *K. pneumoniae* C1559 (left) or *K. pneumoniae*ATCC 33495 (right) and treated with MKM (64 μg/ml, pink) or polymyxin B (25 μg/ml, green) for 6 days. DMSO-treated infected animals (black) showed the lowest survival, whereas MKM and polymyxin B significantly improved survival compared to the DMSO group. Uninfected animals (orange) served as healthy controls. Each curve represents a minimum of three biological replicates (n > 60 worms per group). Survival was monitored daily. Statistical significance was determined using the Log-rank (Mantel–Cox) test.

MKM exhibits selective antimicrobial activity against Gram-negative *Enterobacteriaceae* (*E. coli* and *Klebsiella pneumoniae*) (**Extended Data Table 1;** Fig. 6a, b). It also readily inhibits the growth of mycobacteria. Conversely, MKMs are ineffective against many other Gram-negative and most Gram-positive bacteria (**Extended Data Table 1**). Because the MKM binding site is conserved in ribosomes across various bacterial species, including not only *K. pneumoniae* and *Mycobacterium* sp. (**Extended Data Fig. 8**)^48^, but also the ribosomes from Gram-positive bacteria, such as *Staphylococcus aureus*^49^ (**Extended Data Fig. 8**), the lack of antimicrobial activity of MKM against such strains (**Extended Data Table 1**) is likely due to poor uptake (caused, for example, by the lack of the required transporters), rather than an inability to inhibit translation. Importantly, due to the unique site of action in the ribosome, MKM is insensitive to resistance mechanisms protecting bacterial pathogens from other translation-inhibiting antibiotics (**Extended Data Fig. 9**).

No hemolytic activity or mammalian HEK293T cell toxicity was detected with MKM at a concentration of up to 256 μg/ml (**Table S1**). An *ex vivo* infection model using human blood inoculated with 10^5^ cfu/ml of *K. pneumoniae* C1559 showed complete eradication of bacteria after six hours of exposure to 5×MIC of MKM (Fig. 6c), indicating the potential effectiveness of MKM in treating bloodstream infections. Initial attempts to evaluate MKM in a murine infection model did not yield a strong protective effect, potentially due to limited *in vivo* stability or bioavailability, which will be pursued in follow-up studies. To further assess *in vivo* efficacy, we turned to a *Caenorhabditis elegans* infection model, which offers a convenient and physiologically relevant system for antimicrobial testing. *C. elegans* was infected with *K. pneumoniae* reference strain ATCC 33495 or with the C1559 multidrug-resistant clinical isolate. Infected nematodes were treated with MKM (64 μg/ml), polymyxin B (25 μg/ml), or DMSO, and survival was tracked over six days (Fig. 6d). MKM markedly improved survival in both infection models, with ~55–60% of nematodes remaining alive by day 6, compared to ~10% (ATCC 33495) and ~30% (C1559) in the DMSO-treated groups. Polymyxin B provided a similar survival benefit.

## Discussion

Microbial antibiotic producers may simultaneously produce and secrete several different antimicrobial compounds. In such a scenario, the antibacterial activity of the more moderately expressed inhibitors may be obscured by the most dominant antibiotic. Indeed, *Streptomyces rimosus* has been known as the producer of oxytetracycline for over 75 years and, more recently, as the producer of the Type V glycopeptide antibiotic rimomycin^50^. Only careful fractionation allowed the detection of MKM, a previously unknown antibiotic with an idiosyncratic mechanism of action. It is likely that many other unknown antibiotics may still be hiding in the shadows of the well-studied antibacterials secreted by the well-exploited antibiotic-producing *Streptomyces* strains.

MKM represents a new antibiotic chemical scaffold. The NRPS modules of ManAB predict the production of an Arg-enriched decapeptide corresponding to MKM-E, which we detected in extracts of *S. rimosus* and under MKM BGC heterologous expression in *S. coelicolor*. We further examined the adenylation domains of modules 1, 2, 3, 7, and 8, responsible for Arg activation. Notably, module 3 harbors two mutations in its Stachelhaus^51^ motif (a key region of protein) that correlate with the variable incorporation of Orn or Arg at this position across MKM variants (**Supplementary Fig. 17**), suggesting substrate promiscuity. While rare, such flexibility in adenylation domains has been observed previously in the PheATE module of gramicidin S synthetase, which activates multiple aromatic amino acids^52^.

MKM is the first antibiotic that targets the E site of the large subunit of the bacterial ribosome. The importance of the E site for translation has long been debated^53,54^, and the availability of the large subunit E-site inhibitor MKM will likely serve as a useful tool for investigating the contribution of this functional ribosomal site to the general mechanism of protein synthesis. The binding mode of MKM (MKM-A) determined here is also compatible with other identified MKM congeners, including MKM-B and MKM-E (**Supplementary Fig. 18**), suggesting that these forms are likely to have the same mechanisms of action and inhibit translocation in a context-specific manner as elucidated here for MKM-A.

While several E site inhibitors of eukaryotic or archaeal protein synthesis are known, none of them interfere with bacterial translation due to the structural heterogeneity of the E site across the evolutionary kingdoms (**Extended Data Fig. 6**). In turn, the difference in the ribosomal E site architecture between bacteria and eukaryotes accounts for the therapeutic selectivity of MKM, which is a very weak inhibitor of mammalian translation and does not exhibit toxicity to cultured mammalian cells. Furthermore, because MKM is the first known bacterial large ribosomal subunit E-site inhibitor, none of the ribosome-based resistance mechanisms found in clinical isolates protect sensitive cells from MKM action (**Extended Data Fig. 9**).

The known E-site inhibitors of eukaryotic translation exhibit contrasting modes of action. While CHX arrests the elongating ribosome, structurally related LTM captures the ribosomes at start codons but has little effect on translation elongation or termination^23,29,55^. Superficially, the mode of action of MKM resembles that of CHX, since MKM also stalls the elongating ribosome. However, the mechanism of translation inhibition by CHX and MKM are likely to be principally different. Because of the small size of CHX, it was proposed to co-accommodate in the E site of the eukaryotic ribosome together with the displaced CCA end of the E-site tRNA^23^. This is unlikely to happen with the much bulkier MKM, which nearly completely occludes the E site cavity of the bacterial 50S subunit, thereby blocking the entrance of the CCA end of deacylated tRNA and preventing formation of the P/E hybrid state and translocation (Figs. 3i and 4a).

The severity of translation inhibition by most elongation inhibitors often depends on the sequence of the mRNA or the nascent polypeptide being translated^36^. MKM does not directly interact with mRNA, nor with the growing protein chain. Furthermore, in addition to the ribosome, potential contacts with other players of the translation process are likely to be restricted to the universally conserved CCA ends of E-site tRNAs. Therefore, it was unexpected to find that the bacterial E-site inhibitor still acts in a context-specific manner, more efficiently inhibiting ribosomes translating Pro and Leu codons and exhibiting less effect on those translating Thr codons. While we do not fully understand the structural reasons for the context specificity of MKM action, it is likely related to the kinetics of translation elongation or the duration of the E-site tRNA retention. Exploring further the mechanistic principles of the context specificity of MKM may illuminate unknown aspects of the translation process.

Foreseeing possible mechanisms of resistance necessitates staying ahead in the arms race against bacterial pathogens. Therefore, understanding how bacteria can avoid inhibition by MKM provides us a healthy head start. Our results show that mutations in the peptide transporters provide a low-level of resistance. Altering the chemical properties of MKM, manipulating the overall charge, and making its entrance into the cell independent of the peptide transporters could potentially overcome the uptake-based resistance mechanism and broaden the antimicrobial spectrum. The MKM producer avoids self-toxicity by expressing the ManE rRNA methyltransferase that 2’-O-methylates the 23S rRNA C2395 residue located in the drug binding site (Fig. 5). It is conceivable that if MKM is developed into a drug, the *manE* gene may eventually be acquired by bacterial pathogens. However, our high-resolution structure of the ribosome-MKM complex provides a path for evading the ManE-based resistance by modifying residues of MKM, such as His9, that clash with the C2395 2’-O-methyl (Fig. 5e).

Offering a novel antibiotic scaffold with a new site of action on the bacterial ribosome, combined with activity against challenging Gram-negative pathogens, MKM shows promise for further development. MKM is highly suited for chemical synthesis, allowing for systematic investigation of structure–activity relationships, and efforts to create and assess synthetic analogues are currently in progress. By revisiting ‘old’ producers of antibiotics, we show that there is still a wealth of antibiotic chemical diversity to be mined.

## Materials and Methods

### Bacterial strains and plasmids

Bacterial strains and plasmids used in this study are listed in **Supplementary Table 5**.

### Screening of WAC library for antimicrobial activity

The pre-fractionation library from the Wright Actinomycetes Collection^6^ was screened against hyperpermeable efflux deficient *E. coli* BW25113 Δ*tolC*Δ*bamB* in 384-well microtiter plates (Corning 3701). Each well contained 49 μl of inoculated media (Ca-adjusted Mueller Hinton Broth (MHB; BD Difco^™^) and 1 μl of crude methanolic extract, fractions, or conditioned media (CM). A Biomek FXP Integrated Liquid Handler was used to dispense the fractions, extracts, and inoculated media into the plates. Plates were incubated at 37°C for 20 h in a static incubator. Cell growth was measured by OD at 600 nm using EnVision, SpectraMax, or Biotek Neo microtiter plate readers.

### Purification of manikomycins

*Streptomyces rimosus* WAC 7405 was routinely cultured in TSB media (Difco) in 225 ml flasks for 16 h, before inoculating at 1% (v/v) into ASM medium^56^ in 2.8 l flasks for 4 days. Cultures were maintained at 30°C, shaking at 220 rpm.

During initial discovery, the active compound, as defined by routine testing against *E. coli* BW25113 Δ*tolC*Δ*bamB*, was isolated from conditioned media mixed with 5% (v/v) Diaion HP-20 resin, mixed for 2.5 h. HP20 resin was filtered using a milk filter and extracted with 300 ml of methanol for 2 h. The extract was collected and dried by rotary evaporation. After reconstitution in 10 ml water, compounds were separated by Sephadex LH20 column (300 ml bed volume) and eluted with 50% methanol to yield seven 50 ml fractions. Active fractions were analyzed by using LC-MS/MS method, for this analysis mass range was set to 150–2500 m/z at a scan rate of 1 spectra/sec. Three collision energies of 10, 30, 60 eV was selected with a medium isolation width of 4 amu. The liquid chromatography was performed using a gradient of H_2_O (0.1% formic acid v/v) and acetonitrile on an Eclipse SDB-C8 column (2.1 mm ID × 100 mm, 3.5 μm; Agilent, USA). The flow rate was 0.4 ml/min, and the gradient started with 10% B for 2 min, followed by a linear gradient to 100%B over 15min. After this, the fractions were assessed by Global Natural Products Social Molecular Networking (GNPS)^8^ to identify known compounds. Masses consistent with oxytetracycline were identified in fractions 3, 4 and 5. Fractions 1 and 2 were loaded as a liquid load to reverse-phase Combi Flash column (RediSep Rf C18 High performance Gold-50g, Teledyne Inc) and eluted with a linear gradient of H2O (0.07% trifluoroacetic acid (TFA), solvent A) and ACN (0.07% TFA, solvent B). Active fractions were purified further by preparative reversed-phase high-pressure liquid chromatography (RP-HPLC- Agilent technologies) using C8 column (Eclipse XDB-C8 Semi Prep 9.4×250 mm, 5 μm, Agilent Technologies) with a gradient of 5% to 20% of solvent B in 20 min. MKM-A and MKM-B were eluted in RT 17.5 and RT 18.5 min respectively.

Latter purifications were optimized as follows. Seed cultures in TSB were cultured for 2 days and inoculated into ASM at 10% (v/v). After HP-20 resin, extracts were separated using SP-Sepharose cation-exchange chromatography. The column was pre-equilibrated with a 10 mM ammonium acetate buffer (Buffer A; pH 5.0–5.2). The sample pH was adjusted to the same range. The column was washed with Buffer A and 1 M NaCl in Buffer A at pH 5.0. MKMs were eluted in 1 M NaCl in Buffer A at pH 8.5–9.5. Fractions were neutralized using 0.6 N HCl during the elution. Following combiflash separation, as described, analogues of MKM were resolved on a Chromatik Sunniest RP-Aqua semi-prep column for HPLC (10×250 mm, 5 μm) Purity of the compounds (>95%) was confirmed with a C28 analytical column (Sunniest RP-Aqua C28 4.6×100 mm, 5 μm). MKM-A (most abundant product), MKM-B, and MKM-E were purified as single peaks. Yield varied in different batches of fermentation.

### Structural characterization of MKMs

High-resolution electrospray ionization mass spectra were acquired using an Agilent 1290 UPLC separation module and a qTOF 6550 mass detector in positive ion mode. For general LC separation an Agilent Eclipse XDB C8 column (2.1 × 150 mm; 3.5 μm) and the following method were used: from 0 to 1 min 75% A (0.1 v/v formic acid in water), from 1 to 7 min a linear gradient to 100% B (0.1 v/v formic acid in acetonitrile) at a flow rate of 0.4 ml/min. The NMR spectra were recorded on an AVIII 700 MHz NMR spectrometer, equipped with a cryoprobe. The compounds used in this study were dissolved in deuterated water as a solvent (Cambridge Isotope Laboratories Inc) to a concentration of approximately 5.0 mg/ml. Chemical shifts are reported in ppm relative to TMS using the residual solvent signal at ppm. Chemical shifts values are expressed in ppm (δ), coupling constants (J, Hz) and peak patterns are reported as broad singlet (bs), singlet (s), doublet (d), triplet (t), quartet (q), pentet (p), and multiplet (m). Manikomycin A (1 mg) was treated with 6 N HCl (1 ml) in a sealed tube at 110°C for 24 h. The reaction mixture was then extracted with ethyl acetate and the aqueous solution was dried under nitrogen. To the aqueous residue (10 μl) was added 1 M NaHCO3 (10 μl), followed by Marfey’s reagent (1-fluoro-2,4-dinitrophenyl-5-D-alanine amide) (50 μl, 1% solution in acetone). The reaction was then carried out for 1 h at 40°C and stopped by the addition of 1 N HCl (10 μl) and MeOH (420 μl)^57,58^.

Based on the predicted structure by NMR and HRMS/MS data the following reference amino acids (AA) were selected for modification with Marfey’s reagent: L- and DL-Phe, L- and DL-His, L- and DL-Orn, L- and DL-Thr, L- and DL-Asn and L- and DL-Arg. Briefly, to 10 μl of 10 mg/ml solution of the corresponding AA was added 10 μl of 1 M NaHCO_3_, followed by Marfey’s reagent (50 μl, 1 % (w/v) solution in acetone). The reaction was carried out as described above. The standards and sample hydrolysate were analyzed on LC-MS system (qTOF 6550 coupled to UPLC 1290, Agilent Technologies) with an optimized method to best resolve between individual modified amino acids: 0–0.5 min, 10 % solvent B, 0.5–20 min, linear gradient to 30 %B, 20–40 min linear gradient to 65 %B. Solvent A was 0.1% formic acid in water and solvent B was acetonitrile (100%) at a flow of 0.2 ml/min using an Agilent Eclipse XDB C8 column (2.1×150 mm; 3.5 μm). The absolute configuration of the chiral amino acids was assigned as D-Arg, D-Orn, D-Phe, D-Asn, and L-Thr and L-His.

### Whole genome sequencing and BGC analysis

Genomic DNA extraction and Illumina sequencing were performed as described^59^. The NEB Next Ultra V1 kit was used with 500 ng of sonicated DNA from WAC 7405 and AMPure XP beads were used for size selection. Library preparation and sequencing were performed at the McMaster Genomics Facility. Skewer v0.2.2(2) and FLASH v1.2.11^60^ were used for trimming and merging reads, and SPAdes v3.11.1^61^ for de novo assembly. Illumina assemblies are available from BioProject ID PRJNA1273197. For nanopore sequencing, 400 ng of high molecular weight genomic DNA was prepared with Oxford Nanopore’s Rapid Barcoding Kit and sequenced on a MinION R9.4.1 flow cell. Reads were assembled with Unicycler v0.4.9b^62^ and SPAdes v3.13.0(4). MKM BGCs were identified by analyzing the genome sequence using antiSMASH v6.0.0^63^. The gene annotations of the MKM BGC are shown in **Supplementary Table 2.**

### Heterologous expression of MKM

The MKM BGC was captured using transformation-associated recombination (TAR) cloning^64,65^. DNA sequences were designed targeting MKM BGCs by identifying boundary sequences of the BGCs that included suitable yeast transcription start sites, as outlined in Supplementary Table 3. pCGW^66^ was linearized using NdeI and XhoI restriction enzymes, and the synthesized gBlocks were introduced through Gibson assembly to generate vector MKM-Gbk. The capture vector was linearized with PmeI. Genomic DNA (gDNA) from WAC 7405 was isolated using the salting-out method and treated with RNase A to remove RNA. The purified gDNA was then digested with BstZ17I/XhoI and BstZ17I/XmaJI, sites flanking the MKM BGC. Digested gDNA was further purified via sodium acetate precipitation. Linearized pCGW-gBlocks capture plasmid (~500 ng) and digested gDNA (~2 μg) were co-transformed into *S. cerevisiae* VL6–48N spheroplasts. Captured clones were selected on sorbitol containing SD-trp media (91 g sorbitol (1M), 10 g glucose (2%) and 10 g agar (2%)) containing 0.1% 5-FOA as described in^67^. Positive clones were obtained after 3–5 days at 30°C. Yeast transformants were cultured in SD-trp medium for 24 hours, and plasmid DNA was extracted using the alkaline lysis method for PCR screening. Positive transformants were re-transformed into *E. coli* EPI300 cells by electroporation and confirmed via restriction digestion mapping.

Plasmid pCGW7405 (**Supplementary Fig. 19**) was reintroduced into *E. coli* ET12567 cells via electroporation and mobilized into the host strain *S*. coelicolor M1154 through *E. coli*-*Streptomyces* tri-parental mating, using *E. coli* ET12567/pR9406 as the helper strain^68^ as described^69^.

### Overexpression of ManE rRNA methyltransferase

The ManE gene from the gDNA of the WAC 7405 strain was amplified using primers, METH-TRANS-FP and RP and digested with XhoI and NdeI and cloned into the corresponding sites in pGDP3, a low copy plasmid with a P_lac_ promoter^70^. The corresponding sequence validated plasmid was transformed into *E. coli* BW25113 and BW25113 Δ*tolC*Δ*bamB* strains to assess the impact on MKM susceptibility.

### Minimum inhibitory concentration (MIC) determination

MIC was assessed using the broth microdilution method in cation-adjusted Mueller-Hinton Broth (MHBII, BD Difco) and MOPS minimal medium with 0.4% glucose (M2106 Teknova) following standard procedures unless otherwise specified^71^. Mycobacterial MICs were performed in Middlebrook 7H9 medium supplemented with 10% OADC Enrichment (oleic acid, bovine albumin, dextrose, and catalase) (BD Difco) and 0.05% Tween-80. CFU/ml of all strains were confirmed to be within the range of 1–5 × 10^5^ cells/ml by growth on 7H10+OADC agar. Anaerobic microbiota strains were cultured in BHI supplemented with L-cysteine (0.5 g/l), Hemin (10 mg/l), and Vitamin K (1 mg/l) in anaerobic chambers (37°C, 5% H2, 10% CO2, 85% N2).

### Testing MKM’s cytotoxicity

Mammalian culture was performed in Dulbecco Modified Eagle Medium (DMEM) supplemented with 10% fetal bovine serum (FBS), 2 mM L-glutamine, 100 units/ml penicillin, and 100 μg/ml streptomycin. HEK293 cells (ATCC CRL-1573; generation 6) were seeded at 7500 cells/well in 384-well tissue culture-treated white plates in 50 μl DMEM and incubated for 18h at 37°C in the presence of 5% CO2. After 18 h of incubation, 500 nl of the compound and DMSO (1% final concentration) were added to the cells using a Labcyte Echo acoustic dispenser (Beckman Coulter). Cells were further incubated for 48h and after that cell viability was assessed using Promega Cell Titer Glo 2.0 reagent (Fisher Scientific). Before incubating the plates at room temperature for 10 min, 50 μl of Cell Titer Glo was added directly to the medium. The plates were then shaken for 2 min. The Neo2 plate reader (Biotek) was used to read the luminescence through luminescent fiber. Cells that were either not treated or solely treated with DMSO were used as a control.

### Hemolysis assay

Human blood was obtained from BioIVT (New York, USA). Red blood cells (RBCs) were obtained from blood by centrifuged at 500×g for 5 min. Plasma was removed and the RBCs were washed twice with 150 mM NaCl, using a volume equivalent to the removed plasma. After washing, phosphate-buffered saline (PBS) with a pH of 7.4, in a volume equal to the removed plasma, was added to create the RBC suspension. Using a Labcyte Echo acoustic dispenser (Beckman Coulter), 1 μl of the compound was added to the wells of a 96-well V-bottom plate. The final concentration of DMSO was kept at 1% (v/v), with a DMSO-only negative control included in each replicate. For the positive control, 10 μl of Triton X-100 was added, starting at a concentration of 20% and serially diluted 2-fold to 0.02%. Red blood cells (RBCs) were diluted 1:50 in PBS (pH 7.4), and 99 μl of this diluted solution was added to each well. The plates were incubated at 37°C for 1 h, then centrifuged at 500×g for 5 min to pellet the intact RBCs. 65 μl of the supernatant was transferred to a clear, flat-bottom 96-well plate, and the absorbance was measured at 540 nm.

### Time-dependent killing assay

*E. coli* BW25113 and *Klebsiella pneumoniae* C1559 were grown overnight in 5 ml of cation-adjusted Mueller-Hinton Broth. The cultures were then inoculated into fresh medium to achieve an OD_600_ of 0.1, corresponding to a concentration of 10^8^ CFU/ml. The culture was diluted 100 times, and one ml of the cell suspension was treated with either 5×MIC of MKM-A (160 μg/ml for *E. coli* and 80 μg/ml for *K. pneumoniae*) or tetracycline (5 μg/ml for *E. coli* and 40 μg/ml for *K. pneumoniae*) in a 1 ml culture, incubated at 37°C with agitation at 250 rpm. Samples were taken at 0 h, 3 h, 6 h, and 24 h post-incubation, plated on MHA plates, and incubated for 20–24 hours at 37°C. Colony counts were used to determine the log reduction in CFU/ml, comparing treated strains to untreated control samples.

### Propidium iodide uptake assay

*E. coli* BW25113 cells were prepared according to the method for time-dependent killing and mixed with propidium iodide at a final concentration of 4 μM. A 190 μl aliquot of the cell suspension was added to the wells of a 96-well black-wall plate, followed by the addition of 10 μl of compounds at different concentrations up to 10×MIC (80 μg/ml). Colistin (5 μg/ml) was included as a positive control. Fluorescence was measured at an excitation/emission wavelength of 535/617 nm for 30 min at room temperature using a Synergy Microplate Reader (Biotek).

### Assessing outer membrane permeability by N-Phenyl-1-naphthylamine (NPN) assay

An overnight culture of *E. coli* BW25113 was subcultured into fresh MOPS minimal medium and allowed to grow until reaching mid-exponential phase. The culture was then diluted to an OD _600_ of 0.1–0.2 in the same medium and supplemented with 10 μM NPN dye (prepared from a 20 mM stock solution in acetone). A volume of 190 μl of this cell suspension was combined with 10 μl of test compounds at various concentrations in a black 96-well plate. Water served as the vehicle control, as MKM-A was dissolved in water (adjust the vehicle accordingly based on the solubility of your compound). Colistin at 5 μg/ml was included as a positive control. Fluorescence was measured over a 30 min period at room temperature using a microplate reader, with readings taken every 0.5–1 min at an excitation/emission wavelength of 350/420 nm.

### Scanning electron microscopy

Approximately 10^8^ cells of *E. coli* BW25113 were exposed to MKM at concentrations of 32 μg/ml (5×MIC) and 80 μg/ml (10×MIC) in MOPS minimal medium for 1 h at 37°C. After treatment, the cells were centrifuged at 5,000×g for 5 min, then resuspended in a fixative solution (4% glutaraldehyde in PBS, pH 7.4) at 0.1× the original volume. The cells were fixed at room temperature for 1 h and stored overnight at 4 °C. The following day, 50 μl of the fixed cells were transferred onto coverslips coated with poly-L-lysine, dehydrated through a series of ethanol treatments, and dried using a critical point dryer. The samples were then examined with a scanning electron microscope (TESCAN VEGA-II LSU) equipped with an X-MAX 80mm^2^ EDS detector, and images were captured using INCA software.

### Resistance studies

To develop resistance through sequential passaging, *E. coli* BW25113 cells were grown to OD_600_ of 1–2 and then diluted 200-fold to achieve an OD_600_ of 0.01 in 1 ml of cation-adjusted MHB medium. The cells were incubated at 37°C with shaking in the presence of varying concentrations of MKM-A (0.25×, 0.5×, and 1× MIC) and passaged every 24 hours for 15 days, with the treatment concentration increased daily to promote resistance development. The MIC of the resulting mutants was determined using broth microdilution. Genomic DNA from two resistant mutants (Ecmut1 and Ecmut2) was sequenced using Illumina and analyzed with Breseq v0.37.1 to identify mutations relative to the parental strain^72^.

To select spontaneous mutants, approximately ~10^9^ CFUs of *E. coli* BW25113 and *Klebsiella pneumoniae* C1559 were cultured on cation-adjusted Mueller-Hinton agar, and ~10^9^ CFUs of *E. coli* BW25113 were cultured on MOPS minimal agar plates containing 8×MIC of MKM-A. The plates were incubated at 37°C for 24–48 hours. The resulting colonies were tested for MKM-A susceptibility. The frequency of resistance was determined by dividing the number of colonies on the treated plates by those on the control plates without the compound.

To select resistance mutations in rRNA genes, ~10^9^ CFUs of *E. coli* SQ110D*tolC*::pZ*sbmA* cells^20,21^ overexpressing the transporter involved in MKM uptake were plated on LB agar supplemented with 32 μg/ml (4×MIC) of MKM-A, 100 μg/ml ampicillin, 50 μg/ml kanamycin, 50 μg/ml spectinomycin, and 50 uM IPTG. The plate was incubated overnight at 37°C, the single colonies were randomly picked. The unique 23S rRNA gene (*rrlE*) of this strain was PCR-amplified from 11 clones using the primers 23S_rRNA_F and 23S_rRNA_R and Sanger sequenced.

### In vitro transcription/translation assay

The impact of MKM-A and MKM-E on *in vitro* protein synthesis was evaluated using the *E. coli* S30 extract transcription-translation system (Promega) following the manufacturer’s protocol. The pBESTluc plasmid DNA was utilized as the template to produce firefly luciferase. MKM analogues were tested across a concentration range of 0.05 to 100 μM. The reactions were incubated for one hour at 37°C. Luminescence was then measured in opaque 96-well plates using a Synergy Microplate Reader (Biotek). The IC_50_ values, indicating the concentration at which MKMs inhibited protein synthesis by 50%, were determined using GraphPad Prism 10 software.

### Toeprinting analysis

Toeprinting analysis was carried out in the *E. coli in vitro* transcription-translation system assembled from the purified components (PURExpress, NEB). Toeprinting was carried out using either ^32^P-radiolabeled or fluorescently labeled reverse transcription primers, following the procedures described previously^21,26,27^.

Reactions either contained no antibiotic or were supplemented with 50 μM retapamulin or varying concentrations of MKM. The inhibitors of aminoacyl-tRNA synthetases (mupirocin, Gly-AMS (MedChemExpress: Cat. No.: HY-108940), or Arg-AMS (MedChemExpress, Cat. No.: HY-112862)) were added to the final concentrations of 50 μM to stall translation at downstream “catch codons” (Ile, Gly, or Arg, respectively). The *gltX* template was prepared by amplifying the *gltX* gene from *E. coli* BW25113 genomic DNA using the primers T7_gltX_F and gltX_NV1_R (**Supplementary Table 6**). The *yrbA*_wt template and its derivatives containing 3-Ser or 3-Thr codons were generated by four-primer PCRs using the primers T7_IR_AUG_F, yrbA_wt_F / yrbA_TTT_F / yrbA_SSS_F, yrbA_wt_R/yrbA_Ile_catch_R, and posT-NV1_R (**Supplementary Table 6**).

Fluorescently labeled reactions were carried out on the *ermBL* toeprint mRNA template. The template was generated by PCR of two overlapping 77-nt- and 78-nt-long primers T7_ermBL_F and ermBL_UGA_R (**Supplementary Table 6**). Reactions were assembled in a 6 μl volume with 30 ng of the *ermBL* mRNA template and incubated for 15 min at 37 °C. Reverse transcription was carried out using AMV RT and primer NV*1-Alexa 647 (**Supplementary Table 6**) for 20 min at 37 °C. Reactions were terminated with 1 μl of 5 M NaOH, neutralized with 0.7 μl of 25% HCl, and nucleotide removal was performed with the QIAquick Nucleotide Removal Kit (Qiagen). The samples were dried under vacuum for 2 hours at 60 °C for subsequent gel electrophoresis. The 6% acrylamide gels were scanned on a Typhoon scanner (GE Healthcare). The sequences of all toeprinting templates used in the study can be found in **Supplementary Table 7**.

### Preparation of complexes for structural analysis

MKM-ribosome complexes were generated by *in vitro* translation reactions in the PURExpress In vitro Protein Synthesis Kit (NEB) as described by the manufacturer. Complex formation reactions were carried out on *ermBL* toeprint mRNA template (**Supplementary Table 7**) in a 75 μl of reaction in presence of 50 μM MKM. The reaction was incubated for 15 min at 37 °C. The reaction volume was then split: 69 μl were used for complex generation and 6 μl were further analyzed by toeprinting. Ribosome complexes were isolated by centrifugation in 900 μl of sucrose gradient buffer (containing 40% sucrose, 50 mM HEPES-KOH, pH 7.4, 100 mM KOAc, 25 mM Mg(OAc)_2_ and 6 mM 2-mercaptoethanol) for 3 h at 4 °C with 80,000 *g* in a Optima Max-XP Tabletop Ultracentrifuge with a TLA 120.2 rotor. The pelleted complex was resuspended in Hico buffer (50 mM HEPES-KOH, pH 7.4, 100 mM KOAc, 25 mM Mg(OAc)_2_) supplemented with 50 μM MKM, then incubated for 10 min at 37 °C, similarly to that described previously^26,27^.

### Preparation of cryo-EM grids

Cryo-EM grids were prepared by applying 3.5 μl of MKM-70S complexes onto freshly glow-discharged Quantifoil R3.5/1 grids (copper, 300 mesh, with an additional 3 nm carbon layer; Product: C3-C19nCu30–01). The glow discharge was performed using a GloQube^®^ Plus system (Quorum Technologies) at 25 mA for 30 seconds, in a negatively charged atmosphere. Vitrification of the samples was carried out with a 1:2 ethane-to-propane mixture using a Vitrobot Mark IV (Thermo Scientific). The chamber was maintained at 100% relative humidity and 4 °C. Blotting was performed for 3.5 seconds at blot force 0, using Whatman 597 filter paper. After vitrification, the grids were loaded into autogrid cartridges and stored in liquid nitrogen until further use.

### Data acquisition

Data acquisition was conducted on a Titan Krios G3i transmission electron microscope (Thermo Fisher Scientific/FEI) operating at the Center for Structural Systems Biology (CSSB), Hamburg. The microscope was operated in Fringe-Free Imaging (FFI) mode, equipped with a K3 direct electron detector and a BioQuantum energy filter with a 20 eV slit width. Prior to data collection, gain reference and GIF fine-centering were completed. Automated data acquisition was carried out using EPU software (v3.2.0.4775REL).

Movies were captured at a nominal magnification of 105,000×, corresponding to a calibrated pixel size of 0.832 Å (0.416 Å in super-resolution mode, binned 2× via EPU). The dataset was collected using defocus values ranging from −0.3 μm to −1.0 μm in 0.1 μm increments between holes. Each exposure lasted 1.95 seconds in nanoprobe mode, during which 35 frames were recorded at a dose rate of ~1.14 electrons per frame per Å^2^, resulting in a total accumulated dose of approximately 40 electrons per Å^2^ (~15 e^−^/px/s over vacuum). A 70 μm C2 aperture and beam spot size 7 were used. Objective lens astigmatism was corrected to below 1 nm, and coma-free alignment was refined to under 50 nm using Sherpa’s AutoCTF module (v2.11.1). In total, 5,455 gain-corrected TIFF micrographs of the MKM-70S complex were acquired.

### Cryo-EM data processing

RELION v5.0.0^73,74^ was used for image processing, unless otherwise specified. For motion correction, RELION’s implementation of MotionCor2 with 7×5 patches^75^, and, for initial contrast transfer function (CTF) estimation, CTFFIND version 4.1.14^76^, were employed. Particle picking was performed using crYOLO^77^ and the particle coordinates were then imported into RELION. After 2D classification, all ribosome like particles were selected, extracted with pixel size of 2.49 Å, and 60 Å low pass filtered 70S ribosome (PDB ID 7K00)^28^ was used as reference to perform 3D consensus refinement of these particles. With this 3D refined map, 3D classification was performed without angular sampling. All classes that contained 70S ribosomes at high resolution were used for further processing. Particles with homogenous 3D class distribution were re-extracted using smaller pixel size and subjected to 3D refinements. Subsequently, CTF refinements were performed to correct for anisotropic magnification, defocus and astigmatism, beam tilt, trefoil and higher order aberration followed by Bayesian polishing^78^.

For partial signal subtraction, masks around the region of interest were created. Masking of 3D maps was done using soft mask to avoid artificial correlation and extended to several pixels to avoid overlap with volume. The final 3D refinement was performed focus refining on the 50S, where the drug binds.

After motion correction and CTF estimation, 691,679 particles were picked using crYOLO^77^ (**Fig. Supplementary 11a**). 2D classification with 100 classes was performed and 483,608 ribosome-like particles were selected for further processing (**Fig. Supplementary 11b**). These particles were used as input for consensus refinement against 70S map and then used as input for 3D classification (without angular sampling) (**Fig. Supplementary 11c**). After several rounds of 3D classifications and focus classification on the tRNAs pocket (**Fig. Supplementary 11d-f**), Focus refinement on 50S (70S, P-tRNA, A-tRNA, 261,301 particles) led to final average resolution (gold-standard FSC0.143) of 2.40 Å (**Fig. Supplementary 11g**). 70S containing classes were combined (70S complex, P-tRNA, A-tRNA, 340,201 particles), reaching a final average resolution (gold-standard FSC_0.143_) of 2.45 Å (**Fig. Supplementary 11h**).

### Generation of molecular models

The molecular models were based on the *E. coli* 70S ribosome (PDB ID 7K00)^28^. Starting models with individual chains of ribosomal proteins and rRNA were rigid body fitted using ChimeraX^79^ and modelled using Coot 0.9.8.92^80,81^ from the CCP4 software suite version 8.0^82^. Model refinement was done using Servalcat^83^. For the antibiotic MKM, without available 3D structure, models were generated using ChemDraw (PerkinElmer Informatics) with structural restrains generated using aceDRG^84^. Manual adjustments using real space refinement function was done using Coot^80,81^. The final molecular models were validated using Phenix comprehensive cryo-EM validation tool in Phenix 1.20–4487^85^(**Supplementary Table 4**).

### Figure preparation for cryo-EM data

Particle orientations and their distribution was determined and plotted using Relion v5.0.0^73,74^. The Molprobity server^86^was used to calculate map vs model cross correlation at Fourier Shell Correlation (FSC_0.5_) for all maps (**Fig. S12**). UCSF ChimeraX v1.8^79^ was used to isolate densities, color zone maps and visualize density images. Models were aligned using PyMol version 3.0 (Schrödinger). Figures were assembled using Inkscape v1.3.

### Translocation assay

*In vitro* translocation assay was carried out using the model mRNA MFK (**Supplementary Table 7**) as described in the reference^56^. The mRNA was prepared by *in vitro* transcription of a PCR product amplified using the primers MF_F1, MF_F2, and MF_R (**Supplementary Table 6**). A 4.5 μl reaction containing 1 μM *E. coli* ribosomes, 0.5 μM mRNA, 1 μM tRNA_i_^Met^, 0.5 μM radiolabelled NV1 primer (**Supplementary Table 6**), 2 U/μl RiboLock RNase Inhibitor (Thermo), and antibiotic tested (50 μM MKM or 250 μM negamycin) in Pure System Buffer (PSB; 9 mM Mg(CH_3_COO)_2_, 5 mM K_3_PO_4_, 95 mM potassium glutamate, 5 mM NH_4_Cl, 0.5 mM CaCl_2_, 1 mM spermidine, 8 mM putrescine, 1 mM dithiothreitol, pH 7.3)^87^ was incubated for 20 min at 37°C. Then N-acetyl-Phe-N-tRNA^Phe 88^, in which amino acid is attached to the tRNA’s A3’ hydroxyl via an amide bond, was added to the final concentration of 2 μM followed by 10 min incubation at 37°C. After addition of the *E. coli* EF-G and GTP to the final concentrations of 0.2 μM and 533 μM, respectively and incubation for 5 min at 30°C, 1 μl of the mixture of AMV reverse transcriptase (Roche) and dNTPs (2.1 U/μl AMV RT and 2 mM dNTPs in PSB) was added, and the reactions were incubated for another 5 min at 30°C. The reaction was stopped by addition of 200 μl of the resuspension buffer (300 mM NaCH_3_COO, 5 mM EDTA, 0.5% SDS), DNA was then isolated by phenol-chloroform extraction, precipitation by addition of 3 volumes of ice-cold ethanol, incubating at −70°C for 15 min, and centrifugation for 30 min (4°C, 20000 g). The reaction products were resolved in 6% sequencing polyacrylamide gel and imaged on the Typhoon phosphorimager.

### Semiquantitative analysis of methylated 23S ribonucleoside abundance

To identify the posttranscriptional modification installed by ManE 50S ribosomal subunits were isolated from *E. coli* BW25113 transformed with either empty vector pGDP3^70^or pGDP3-*manE*, constitutively expressing ManE methyltransferase under the control of the P_bla_ promoter. Overnight cultures of the two strains were diluted 1:50 in 75 ml of LB medium supplemented with 100 mg/ml ampicillin and incubated with shaking for 5 h (37°C, 240 rpm). The cultures were chilled on ice for 10 min, and the cells were collected by centrifugation at 4400 g for 10 min at 4°C. Cell pellets were washed once with 20 ml of Wash Buffer (50 mM HEPES pH 7.6, 10 mM MgCl_2_, 50 mM NH_4_Cl), frozen in liquid nitrogen, and stored at −80°C.

For isolation of the ribosomes, 0.5 g of frozen cell paste for each strain was resuspended in 0.7 ml of Lysis Buffer (20 mM Tris/HCl pH 8.0, 10 mM MgCl_2_, 100 mM NH_4_Cl, 5 mM CaCl_2_, 0.4% Triton X-100, 0.1% NP-40, 1 mg/ml lysozyme, 100 U/ml DNAse I (Roche), 320 U/ml SUPERase·In RNase Inhibitor (Invitrogen)) and incubated on ice for 30 min. Cell suspension was transferred into three 2-ml tubes containing 400 mg of Lysing Matrix B beads (MP Biomedicals) each. Cells were lysed in FastPrep-24^™^ bead beater (MP Biomedicals) (3 min, 6.5 beats/sec). The tubes were centrifuged 12 min at 20 000 g (4°C) and 600 ml of clarified lysates were layered on top of 1.7 ml of sucrose cushion (20% sucrose, 20 mM Tris/HCl pH 8.0, 10 mM MgCl_2_, 100 mM NH_4_Cl) in the tubes for the S110AT rotor of Sorvall MX 120 Plus Micro-Ultracentrifuge (Thermo). Ribosomes were pelleted by centrifugation at 422,000 × g for 1 h (4°C). The ribosome pellets were rinsed with 300 μl of Resuspension Buffer (20 mM Tris/HCl pH 8.0, 1.5 mM MgCl_2_, 100 mM NH_4_Cl) and then resuspended in 300 μl of the same buffer. The samples were centrifuged at 20, 000 × g for 10 min (4°C) and 70 A_260_ units from each sample were loaded on top of two tubes (12 ml each) with 5–20% sucrose gradients prepared in the following buffer: 20 mM Tris/HCl pH 8.0, 1 mM MgCl_2_, 100 mM NH_4_Cl. Tubes were centrifuged for 2.5 h at 273,000 g (39, 000 RPM) in SW 41 Ti rotor (Beckman). The contents of the tubes were fractionated using piston gradient fractionator (Biocomp Instruments) and the fractions containing 30S ribosomal subunits were collected.

Total RNA was isolated from the fractions by hot phenol/chloroform extraction procedure as follows: acid-phenol : chloroform : isoamyl alcohol pH 4.5 (125:24:1, Ambion) prewarmed to 65°C was added to sucrose gradient fractions in 1:1 ration (v/v) and the mixture was incubated for 5 min at 65°C with shaking (1400 rpm) followed by centrifugation at 15,000 × g for 2 min. The aqueous phase was transferred to a new tube and phenol extraction was repeated with 1 vol of room temperature acid-phenol: chloroform: isoamyl alcohol mixture. After that 0.9 vol of chloroform was mixed with aqueous phase followed by immediate centrifugation. The RNA from aqueous phase was then precipitated by addition of NaOAc, pH 5.5, to the final concentration of 300 mM and 1.1 vol of ice-cold isopropanol followed by 30 min incubation at −80°C. RNA was pelleted by centrifugation at 20,000 g 30 min (4°C) and supernatant was discarded. The RNA pellet was rinsed with 0.8 ml of ice-cold 80% ethanol and then resuspended in 60 μl of 10 mM Tris/HCl pH 7.0. The quality of the 23S rRNA was analyzed by agarose gel electrophoresis and the presence of C2395 modification was confirmed by primer extension (see the Primer extension section below).

25 μg of RNA from each sample were digested overnight by 1 U of Nuclease P1 (NEB) at 37°C in 50 μl reactions containing 1x P1 Reaction Buffer (NEB) supplemented with 0.8 mM ZnSO_4_. In order to convert the resulting ribonucleotides to ribonucleosides, 0.25 U of Shrimp Alkaline Phosphatase (rSAP, NEB) and 5.5 μl of 10 × rSAP Buffer (NEB) were added and the reactions were incubated at 37°C for 3 h.

Samples containing ribonucleosides were further purified by extraction with 90% acetonitrile:water to remove insoluble material and concentrated by vacuum centrifugation. Samples were dissolve in 90% acetonitrile:water and analyzed by high-resolution LC-MS on an Agilent 6546 LC-Q-TOF by hydrophilic interaction chromatography according to published methods^89^.Samples were separated on an Agilent Poroshell 120 HILIC-Z column (2.7 μm, 2.1×150 mm) at 0.1 ml/min in a 10 mM ammonium acetate (pH 5.2)-acetonitrile gradient starting with 10% of acetonitrile to 60% in 32 min, followed by 4 min isocratic and returning to 10% in 1 min with isocratic again at 6 min and further 6 min post run. The flow-rate was set to 0.1 ml/min. Retention times of methylated cytidine and guanosine nucleoside standards were defined using compounds obtained from Cedarlane (Cm, 5mC, Gm, 1mG, 7mG) and TargetMol (m2G).

Integrated ion intensities were determined for hydrogen, sodium, and potassium adducts of expected nucleosides, methylated nucleosides, and nucleobases produced through in-source fragmentation. Mass error for all analyzed nucleoside ions was less than 5 ppm. Signals for all ions were normalized by the median intensity and compared between control or methyltransferase containing *E.coli* cells to identify ions with 1.5-fold or greater change in intensity.

### Primer extension

Total RNA was extracted from the corresponding strains of *E. coli* using the RNeasy total RNA extraction kit (Qiagen). Primer extension analysis of rRNA modifications was performed using one microgram of total RNA essentially as described in^90^. Primer L2507 (**Supplementary Table 5**) was used for the analysis of C2395 modification.

### Ribosome profiling

Ribo-seq experiments were performed as described previously^90^. Briefly, the overnight culture of *E. coli* BL21D*tolC* was diluted 1:100 in 4 flasks (2 MKM-treated and 2 control samples) containing 100 ml of MOPS minimal medium (M2106 Teknova) each. The cultures were grown at 37°C until reaching the OD_600_ of ~0.55. For MKM-treated samples, MKM-A dissolved in DMSO was added to the cultures to a final concentration of 50 μg/ml (25×MIC), and incubation continued for 2 min. Equivalent amount of DMSO was added to control samples for 2 min. Cells were harvested by rapid filtration and flash frozen in liquid nitrogen. Cell pellets were resuspended in 300 μl of cold lysis buffer (20 mM Tris, pH 8.0, 10 mM MgCl_2_, 100 mM NH_4_CL, 5 mM CaCl_2_, 0.4 % Triton-X100, and 0.1% NP-40) supplemented with 3 mM GMPPNP, 30 U RNase-free DNase I (Roche) and 96 U Superase•In^™^ RNase inhibitor (Invitrogen) and lysed by bead-beating with 300 mg of zirconium beads in the FastPrep-24 bead-beater (MP Biomedicals) for 1 min at 6.5 beats s^−1^. Cell lysates were clarified by centrifugation at 20,000×g for 10 min at 4°C. 22 A_260_ units of the clarified lysates were treated with 880 U of *S. aureus* Micrococcal Nuclease (MNase, Roche) for 60 min at 25°C. The MNase reaction was quenched by addition of EGTA to final concentration of 6 mM. The lysates were layered over 2 ml of sucrose cushion (20% sucrose, 20 mM Tris/HCl pH 8.0, 10 mM MgCl_2_, 100 mM NH_4_Cl) in 4 ml tubes for S110AT rotor of Sorvall MX 120 Plus Micro-Ultracentrifuge (Thermo Fisher). Ribosomes were pelleted by centrifugation for 1 h at 422,000×g (100,000 RPM). The pellets were resuspended in 500 μl of resuspension buffer (20 mM Tris/HCl pH 8.0, 10 mM MgCl_2_, 100 mM NH_4_Cl, 1% SDS) and frozen in liquid nitrogen. Subsequent isolation of ribosomal footprints and library preparation were performed as described in^91^.

A custom script was used to demultiplex the samples, remove the linker barcode and then remove 5 nts from the 3’ end and 2 nts from the 5’ end, which were added as part of the library design^91^. Bowtie2 (v2.2.9)^92^ within the Galaxy pipeline first aligned the trimmed reads to the non-coding RNA sequences. The remaining unmapped reads were aligned to the reference genome of the *E. coli* strain BL21 (GenBank ID CP053601.1). The 24 to 46 nt-long reads were used in the subsequent analyses. The first position of the P-site codon was assigned 15 nt from the 3’ end of the read^37^.

The metagene analyses at the annotated start and stop regions followed the described protocol^93^. Included in the analysis were the ORFs that were: a) separated by at least 50 nt; b) with the length of 300 nt or more; c) with at least 20% of the positions had assigned reads values above zero; d) with the average number of RPM per nt ≥ 0.005. For the metagene plots, ribosome footprint density was normalized to the average coverage of the ORF including 50 flanking nts. The mean of the normalized values was computed and plotted for the ORF segments around the start and stop codons.

To analyze sequence specificity of MKM-induced ribosome stalling we first selected the codons in the bodies of the genes (excluding the first 10 and last 3 codons of the genes), for which the ribosome occupancy was at least 5 times higher in the MKM-treated sample compared to the control (data from duplicates were merged for this analysis). For each site, the corresponding sequence of the amino acids was determined, and the over- or underrepresentation of amino acids for each position around the stall was analyzed using the online pLogo tool^94^ (https://plogo.uconn.edu/) with selected (n = 5248) and total (n = 194,045) samples of stalling sequences.

### *Ex vivo* efficacy

Human blood was procured from BioIVT (New York, USA). *Klebsiella pneumoniae* C1559 cells were cultivated in cation-adjusted Mueller-Hinton media until the OD_600_ reached 1. Then, 10 μl of these cells were introduced into 990 μl of blood with 5×MIC of MKM-A. Control cells, which did not receive treatment, were cultured on MHB agar alongside the treated samples after incubation periods of 0 h, 3 h, and 6 h at 37°C. CFUs were determined by serial dilution and cultivation on agar.

### *C. elegans – K. pneumoniae in vivo* antibiotic activity assay

A C. elegans double mutant strain, AU37 *(glp-4(bn2);sek-1(km4))*, was used for this assay due to its enhanced pathogen infection sensitivity and temperature-sensitive sterility. The infection protocol was carried out as previously described^95^, with slight modifications to accommodate our experimental conditions. Standard *C. elegans* media and protocols were used for the maintenance and growth of worms^96^. To summarize, eggs were collected from gravid adult worms by bleaching and incubated on solid agar plates at 25 °C for 48 hours, until early adulthood. Worms were thoroughly washed with M9 buffer and transferred onto LB agar plates containing lawns of infective *K. pneumoniae* and incubated for another 24 hours. Worms were then washed from plates and resuspended in M9 to an approximate 2 worm per microlitre concentration. A survival assay was conducted in a 96-well plate with experimental wells containing 15 μl of worms, 80 μl S-basal (5.85 g NaCl, 1 g K_2_HPO_4_, 6 g KH_2_PO_4_, 1 ml cholesterol (5 mg/ml in ethanol), H_2_O to 1 litre. Sterilize by autoclaving), and 5 μl of test compound. Plates were sealed with a porous film and incubated at 25°C for a total of 7 days. The number of dead worms were counted every 24 hours to generate a Kaplan-Meier survival curve.

### Identification of ManE-containing BGCs and phylogenetic tree construction

The protein sequence of ManE was used as a query in NCBI BLASTp. The results were manually curated to obtain non-redundant protein sequences. To analyze the corresponding genomes that showed the presence of *manE* homologs, antiSMASH v.7.0 was employed. The BGCs from each strain were extracted and compared using Clinker^97^. The manE-like methyl transferases from these BGCs were extracted and aligned with the in-built MUSCLE algorithm in MEGA11^98^ using default settings. This alignment was employed to build a maximum-likelihood (ML) phylogenetic tree with MEGA11, utilizing the WAG substitution model, a bootstrap value of 100, and default parameters. An unrelated RlmE methyltransferase from *E. coli* K12 was used as an outgroup for tree rooting.

### Statistical methods

Unless otherwise stated in the figure legend, a two-way ANOVA was used for multiple comparisons, and a Log-rank (Mantel–Cox) test was used for survival curve analysis. Values were considered statistically significant at p ≤ 0.05, p ≤ 0.01, or p ≤ 0.001, as indicated in the figure legends. All data were collected and organized using Microsoft Excel and statistical analyses were performed with GraphPad Prism 10.2.3. and v. 10.5.0.

## Supplementary Material

This is a list of supplementary files associated with this preprint. Click to download.

- SUPPLEMENTARYDATAFIGURES.docx
- EXTENDEDDATAFIGURESandTable.docx
- VideoS1.mp4

## Data Availability

Cryo-EM maps and molecular models were deposited in the Electron Microscopy Data Bank (EMDB) and Protein Data Bank (PDB) with accession codes EMD-54009 and PDB ID 9RJA (MKM-70S complex) and EMD-53943 and PDB ID 9RFW (MKM-50S complex), respectively. All previously published structures used in this work for structural comparisons were retrieved from the RCSB Protein Data Bank: PDB entries 8GLP, 6SGC, 4U3U, 2OTJ, 4U4R, 4U4Z 5O61, 8P2G, 6SPG, 6ND6. The complete genome sequence of *Streptomyces rimosus* WAC 7405 is deposited in NCBI GenBank with BioProject ID PRJNA1273197. Sequencing data collected for ribosome profiling experiment were deposited in NCBI Sequence Read Archive (SRA) with BioProject ID PRJNA1265262. All other materials used in this study are available from corresponding authors upon request.
